# IgG1 and IgG4 Antibody Responses to the *Anopheles gambiae* Salivary Protein gSG6 in the Sympatric Ethnic Groups Mossi and Fulani in a Malaria Hyperhendemic Area of Burkina Faso

**DOI:** 10.1371/journal.pone.0096130

**Published:** 2014-04-23

**Authors:** Cinzia Rizzo, Raffaele Ronca, Fabrizio Lombardo, Valentina Mangano, Sodiomon Bienvenu Sirima, Issa Nèbiè, Gabriella Fiorentino, Marita Troye-Blomberg, David Modiano, Bruno Arcà

**Affiliations:** 1 Department of Public Health and Infectious Diseases - Parasitology Section, “Sapienza” University, Rome, Italy; 2 Department of Biology, “Federico II” University, Naples, Italy; 3 Centre National de Recherche et de Formation sur le Paludisme, Ouagadougou, Burkina Faso; 4 Department of Molecular Biosciences, The Wenner-Gren Institute, Stockholm University, Stockholm, Sweden; 5 Istituto Pasteur Fondazione Cenci-Bolognetti, Sapienza University, Rome, Italy; University of Notre Dame, United States of America

## Abstract

Human antibody response to the *Anopheles gambiae* salivary protein gSG6 has recently emerged as a potentially useful tool for malaria epidemiological studies and for the evaluation of vector control interventions. However, the current understanding of the host immune response to mosquito salivary proteins and of the possible crosstalk with early response to *Plasmodium* parasites is still very limited. We report here the analysis of IgG1 and IgG4 subclasses among anti-gSG6 IgG responders belonging to Mossi and Fulani from Burkina Faso, two ethnic groups which are known for their differential humoral response to parasite antigens and for their different susceptibility to malaria. The IgG1 antibody response against the gSG6 protein was comparable in the two groups. On the contrary, IgG4 titers were significantly higher in the Fulani where, in addition, anti-gSG6 IgG4 antibodies appeared in younger children and the ratio IgG4/IgG1 stayed relatively stable throughout adulthood. Both gSG6-specific IgG1 and IgG4 antibodies showed a tendency to decrease with age whereas, as expected, the IgG response to the *Plasmodium* circumsporozoite protein (CSP) exhibited an opposite trend in the same individuals. These observations are in line with the idea that the *An. gambiae* gSG6 salivary protein induces immune tolerance, especially after intense and prolonged exposure as is the case for the area under study, suggesting that gSG6 may trigger in exposed individuals a Th2-oriented immune response.

## Introduction

The ability of hematophagous insects to feed on a protein-rich source such as blood involves complex behavioral, morphological and physiological adaptations to find suitable hosts, reach blood vessels and to suck and digest blood. One of the results of these adaptations to blood feeding was the evolution of repertoires of salivary proteins playing crucial functions in counteracting the hemostatic, inflammatory and immune responses of vertebrate hosts to tissue injury [1]. These proteins, injected into the skin during the blood meal, play essential roles in blood feeding but also trigger an anti-saliva antibody response that can be exploited as a tool to evaluate host exposure to disease vectors as diverse as ticks [2], sandflies [3], triatomines [4], tsetse flies [5], [6] and mosquitoes [7]–[11]. Transcriptome studies during the last five to ten years allowed to unravel the complexity of the salivary repertoires of different mosquito species establishing that they carry in their saliva around 70 to 130 salivary proteins [12]–[14]. Moreover, comparative analyses identified genus-specific proteins and protein families, which are found for example in the saliva of *Anopheles* mosquitoes but are absent in *Aedes* and *Culex* species, or viceversa [15]. These genus-specific proteins, if immunogenic, may represent ideal candidates for the development of sensitive, reliable and reproducible serological tools for the evaluation of human exposure to vectors of important human diseases such as malaria or dengue.

Evaluation of malaria transmission and disease risk requires both parasitological and entomological measurements, with the latter classically based on the Entomological Inoculation Rate (EIR) that is the number of infectious bites per person per unit of time. However, determination of EIR can be difficult or impossible in several epidemiological settings (low malaria transmission, low or reduced vector density, logistic problems, etc.) as well as in children (where assessment of *Anopheles* exposure by human landing catches is ethically unfeasible). Thus, alternative tools would be extremely valuable. In this respect the *Anopheles gambiae* gSG6 salivary protein appears a very promising tool for the evaluation of human exposure to malaria vectors. gSG6 is a small anopheline-specific protein which is exclusively expressed in adult female salivary glands, it is relatively abundant in saliva and plays a role in blood feeding [16], [17]. Previous studies on populations from Burkina Faso [18], Tanzania [19] and Uganda [20] showed that human IgG response to the gSG6 protein is sufficiently short lived to detect variation in exposure to malaria vectors both in time and in space. Moreover, the anti-gSG6 IgG response to the *An. gambiae* protein also reflects exposure to *Anopheles arabiensis* and *Anopheles funestus* and, therefore, it may be considered as a reliable indicator of human exposure to all three main Afrotropical malaria vectors [21]. Using the gSG6-P1 peptide, which is designed on the gSG6 protein, similar results were obtained by Remoue and collaborators [22], [23], who also showed it may be a valuable tool to evaluate the efficacy of malaria vector control interventions, such as the application of Insecticide Treated Nets [24], [25]. Considering that SG6 family members (i) are widespread among anophelines (only exception so far appear to be Central and South American species of the subgenus *Nyssorhynchus*) and (ii) are well conserved in comparison to gSG6 (amino acid identity from 52% with *Anopheles dirus* to 100% with members of the *An. gambiae* complex) it is likely that the gSG6 protein may represent a relevant indicator of human exposure to a wide range of anopheline species.

We previously analyzed the response to the gSG6 protein in Mossi and Fulani, two sympatric ethnic groups from a malaria hyperendemic area of Burkina Faso. These two groups are known for their different susceptibility to malaria, with the Fulani being less parasitized, less affected by the disease and more responsive to parasite antigens [26], possibly as a consequence of a functional deficit of T regulatory cells (Treg) [27]. In our study the Fulani also exhibited a higher IgG response to the *An. gambiae* salivary protein gSG6. However, while a seasonal variation of the anti-gSG6 IgG response was found in the Mossi, no significant difference between high-transmission/rainy season and low-transmission/dry season was observed in the Fulani [18]. Moreover, in both ethnic groups the IgG response to the gSG6 antigen was higher in young children and tended to decrease in adults, suggesting that the intense and prolonged exposure to bites of anopheline mosquitoes may induce immune tolerance toward this antigen [18]. We report here the analysis of IgG1 and IgG4 subclasses among anti-gSG6 IgG responders of the Mossi and Fulani ethnic groups from Burkina Faso.

## Materials and Methods

### 2.1. Study Area and Subjects

The study was conducted in the villages of Barkoundouba and Barkoumbilen, which are located at a five kilometers distance from each other in a rural malaria hyperendemic area of Burkina Faso (∼35 kilometers NE of Ouagadougou). These two villages are inhabited by two ethnic groups with different immune reactivity and susceptibility to *P. falciparum* malaria, with the Fulani living in Barkoundouba and the Mossi in Barkoumbilen [26]. The area is characterized by intense *P. falciparum* transmission, especially during the June–October rainy season (EIR>100/person/year). Malaria prevalence is very high, and ∼95% of malaria infections are by *P. falciparum*. In the high transmission season infection rates range from 60% to 90% (depending on age) in the Mossi-Rimaibé group, and from 20% to 80% amongst Fulani. Lower prevalence is observed during the dry, low transmission season (40–80% amongst Mossi and Rimaibé and 0–60% in Fulani). Sera were collected during three cross-sectional surveys carried out at the beginning (August 1994) and the end (October 1994) of the high-transmission/rainy season, as well as during the following low-transmission/dry season (March 1995). *Plasmodium falciparum* inoculation rates were comparable in the two villages as previously reported [28].

### 2.2. Ethics Statement

The study protocol was approved by the Technical Committee of the Centre National de Lutte contre le Paludisme of the Ministry of Health of Burkina Faso. Oral informed consent for multiple immuno-parasitological, clinical and entomological surveys was obtained from both the Fulani community living in Barkoundouba and the Mossi community living in Barkoumbilen. The samples utilized for this analysis have been collected in the period from August 1994 to March 1995 in the frame of a larger epidemiological study performed in Burkina Faso [26]. At that time a national ethic committee was not yet in place and written consent was not requested. Therefore, the study protocol and the oral informed consent were approved by the Institutional Review Board, i.e. by the Technical Committee of the Centre National de Lutte contre le Paludisme of the Ministry of Health of Burkina Faso. Individual oral consent was obtained from all adults and from children’s parents or legal representatives. In June–July 1994, prior to the start of the surveys, meetings were held in the villages of the study area to explain, in the local languages, the objectives of the study, the procedures involved and to answer questions from the residents.

### 2.3. Entomological Data

Entomological measures were based on indoor pyrethrum spray catches carried out monthly between August and November ‘94 and in March ‘95 (12 catches/month). In the study area the main malaria vectors were *An. gambiae*, *An. arabiensis* and *An. funestus*, with the members of the *An. gambiae* species complex (i.e. *An. gambiae* and *An. arabiensis*) representing, on average, approximately 90% of the indoor-resting *Anopheles* mosquitoes. The number of anopheles/person/night in the two villages under study has been previously reported [18] and additional details on the study site and parasitological aspects can be found elsewhere [26], [28].

### 2.4. Plasma Samples

A total of 270 independent human sera (148 Mossi, 122 Fulani) selected among anti-gSG6 IgG responders have been analyzed here. These responders were identified in a previous study [18] using as cut-off value for seropositivity the mean OD of unexposed controls (sera from 60 Roman citizen) plus 3xSD. The collection time, the number of individuals and ethnic group and the average age in years ± the 95% confidence interval (CI) were as follows. August: 60 Mossi (22.0±4.5), 63 Fulani (16.1±3.9); October: 56 Mossi (13.0±3.4), 30 Fulani (21.2±7.0); March: 32 Mossi (15.3±5.1), 29 Fulani (11.6±4.2). Sera from 44 Roman citizen (1–81 years old, 33.1±7.1) who referred to a city hospital for routine blood tests were used as control (unexposed individuals). Data on IgG response to the *P. falciparum* circumsporozoite protein (CSP) of the same 270 anti-gSG6 IgG responders and of 28 Roman citizen used as control were from a previous larger study [26].

### 2.5. Enzyme-linked Immunosorbent Assays

ELISA was performed according to standard procedures. The gSG6 protein was expressed and purified as previously described [18]. Flat-bottom, 96-well plates (Nunc Maxisorp, M9410) were coated overnight with 50 µl of gSG6 at 10 µg/ml diluted in coating buffer (15 mM Na_2_CO_3_, 35 mM NaHCO_3_, 3 mM NaN_3_, pH 9.6). Wells were washed four times with PBST (0.05% Tween-20 in 1x PBS), blocked for 3 hours at 25°C (150 µl 1% w/v skimmed dry milk in PBST), washed again as above and incubated overnight at 4°C with 50 µl of serum diluted 1∶20. Plates were then washed as above and incubated (3 hours, 25°C) with 100 µl of sheep anti-human IgG1/HRP (Binding Site AP006) or 100 µl of sheep anti-human IgG4/HRP (Binding Site AP009) both diluted 1∶1000 in blocking buffer. After washing the colorimetric reaction was carried out with 100 µl of o-phenylenediamine dihydrochloride (OPD, Sigma P8287; 15 min, 25°C in the dark). Reactions were terminated by adding 25 µl of 2 M H_2_SO_4_. OD_492_ were determined using a microplate reader (Biotek Synergy HT).

IgG1 and IgG4 OD levels were converted to antibody titers using standard curves set up as follows. As capturing factors goat anti-human IgG (5 µg/ml; Jackson ImmunoResearch Laboratories Inc, PA, USA) or mouse anti-human IgG4 (2 µg/ml; BD Pharmingen, USA) were used for coating (50 µl coating buffer, overnight at 4°C). After washing, blocking and washing again as above wells were incubated overnight at 4°C with serial dilutions, from 1 µg/ml to 0.0078 µg/ml (1 → 0.5 → 0.25 → 0.125 → 0.0625 → 0.03125 → 0.0156 → 0.0078), of purified native human IgG1 or IgG4 (ABD Serotec, Kidlington, Oxford, UK) in 50 µl of blocking reagent. Incubation with anti-human IgG1/HRP or IgG4/HRP and colorimetric detection were as described above. The CSP IgG antibody response was evaluated with an ELISA kit based on (NANP)_40_ antigen as previously described [26].

### 2.6. Data Analysis

IgG1 and IgG4 levels were determined by analyzing serum samples in duplicate with the antigen and once without antigen (coating buffer only). Final OD was calculated for each serum as the mean OD value with antigen minus the OD value without antigen. Serial dilutions of a pool of sera (1∶6 → 1∶18 → 1∶54 → 1∶162 → 1∶486 → 1∶1458 → 1∶4376) were added to each plate as standard curve to normalize experimental variability among plates. Intra and inter assay variation of standard samples was below 20%. Sera whose duplicates showed a coefficient of variation >20% were not included into the analysis. Multiple comparisons were performed by the Kruskal-Wallis test. Mann-Whitney U test was used to compare IgG, IgG1 and IgG4 levels among responders of two independent groups. The Wilcoxon matched-pairs test was used for comparison of two paired groups. Correlation was assessed by Spearman coefficient. All statistical analyses were performed using GraphPad Prism 5.0 statistical software (GraphPad Software Inc., La Jolla, CA).

## Results

The IgG1 and IgG4 antibody response to the *An. gambiae* gSG6 protein was measured in 270 human sera collected in the villages of Barkoundouba and Barkoumbilen, which are inhabited by the Fulani and Mossi ethnic groups, respectively. Sera were collected during three cross-sectional surveys, carried out at the beginning (August) and the end (October) of the high-transmission/rainy season as well as during the following low-transmission/dry season (March), and they were selected among anti-gSG6 IgG responders identified in a previous study [18]. As a comparison, data on the IgG response to the *P. falciparum* CSP in the same 270 anti-gSG6 IgG responders were retrieved from a previous larger study [26]. EIR were comparable in the two villages as previously reported [28], although the number of *Anopheles*/person/night, as measured by the indoor pyrethrum spray catch method, was slightly higher in Barkoundouba. Anyhow, a drop in anopheline density was clearly evident in both villages during the dry season [18].

### 3.1. IgG1 and IgG4 Antibody Response to the *An. gambiae* gSG6

**Figure 1:**
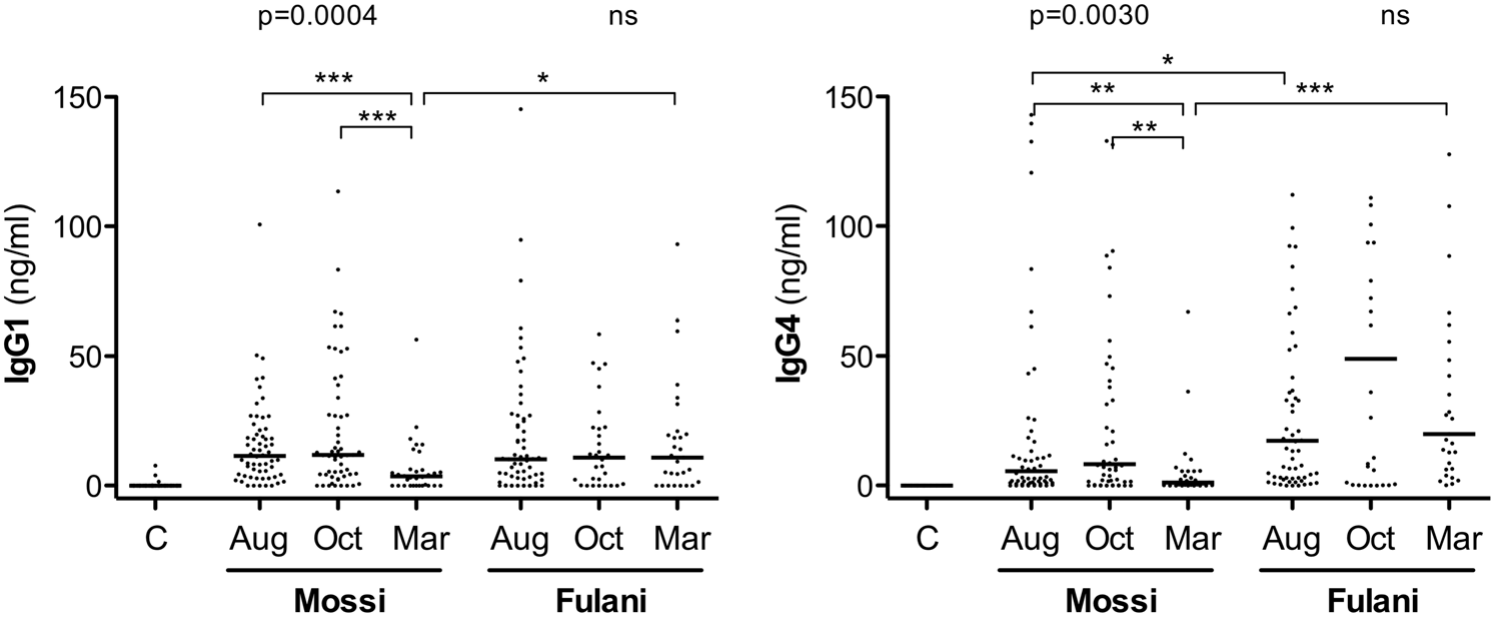
Seasonal variation of the gSG6-specific IgG1 and IgG4 antibody titers in the sympatric ethnic groups Mossi and Fulani. Scatter plots reporting IgG1 (left panel) and IgG4 (right panel) antibody titers among gSG6 IgG responders of the Mossi and Fulani ethnic groups in the three different surveys. C, unexposed controls. Bars indicate median values. Number of individuals analyzed (n) and average age in years ±95% CI were as follows. August: Mossi n = 60 (22.0±4.5), Fulani n = 63 (16.1±3.9); October: Mossi n = 56 (13.0±3.4), Fulani n = 30 (21.2±7.0); March: Mossi n = 32 (15.3±5.1), Fulani n = 29 (11.6±4.2); Controls n = 44 (33.1±7.1). P values determined according to the Kruskal-Wallis test. Pairwise comparisons refer to the Mann-Whitney U test (*, 0.01<p<0.05; **, 0.001<p<0.01; ***, p<0.001). Note that one (left panel) and twenty-six (right panel) data points are outside the axis limits.

When the anti-gSG6 IgG1 and IgG4 antibody titers were compared among the different surveys, a seasonal variation according to the high-transmission/rainy season was observed in the Mossi (Kruskal-Wallis test: IgG1, p = 0.0004; IgG4, p = 0.0030), whereas it was absent in the Fulani (Figure 1). More specifically, in individuals from the Mossi ethnic group both IgG1 and IgG4 significantly decreased during the dry season (Mann-Whitney U test, p≤0.0005 and p≤0.0045, respectively); on the contrary, no significant differences were found in the Fulani, although the median IgG4 value showed a peak at the end of the transmission season (Figure 1). These observations are consistent with previous findings showing seasonal variations of the anti-gSG6 IgG response in the Mossi but no difference in the Fulani [18].

The anti-gSG6 IgG1 antibody titers were comparable between the two ethnic groups: only during the dry season, when the antibody response drops among Mossi, IgG1 levels were higher in the Fulani (Figure 1 and Table 1; Mann-Whitney U test, p = 0.0200). On the contrary, median IgG4 levels were consistently higher in the Fulani and this difference was significant in August and March (Figure 1 and Table 1; Mann-Whitney U test, p = 0.0206 and p<0.0001, respectively). Moreover, when the IgG1 and IgG4 responses were compared in the same group of individuals from the different surveys and ethnic groups anti-gSG6 IgG4 antibodies appeared as the dominant subclass in the Fulani; instead, no difference was found in the Mossi where, furthermore, median IgG4 titers were consistently lower than corresponding IgG1 titers (Table 1).

**Table 1:** Comparison of the gSG6-specific IgG1 and IgG4 antibody titers in individuals of the Mossi and Fulani ethnic groups in the different surveys.

Survey	Group^1^	IgG1^2^	IgG4^2^	P-value^3^
**Aug ‘94**	**M**	**11.49** (3.88–20.97)	**5.56** (0.98–24.32)	**ns**
**Oct ‘94**	**M**	**11.80** (4.24–32.31)	**8.25** (1.45–54.37)	**ns**
**Mar ‘95**	**M**	**3.53** (0.00–6.24)	**1.12** (0.00–5.55)	**ns**
**Aug ‘94**	**F**	**10.18** (2.16–27.04)	**17.24** (2.99–58.96)	***
**Oct ‘94**	**F**	**10.87** (0.48–22.45)	**48.88** (0.42–108.80)	***
**Mar ‘95**	**F**	**10.79** (0.70–20.40)	**19.86** (6.30–58.80)	**
**-**	**C**	**0.00** (0.00–0.00)	**0.00** (0.00–0.00)	**ns**

**(1)**. M. Mossi; F, Fulani; C, Controls.

**(2)**. Median antibody titer (ng/ml); 25^th^–75^th^ percentiles are given in parentheses.

**(3)**. Pairwise comparison by the Wilcoxon matched-pairs signed rank test. Number of individuals, average age and asterisks as in Figure 1.

### 3.2. IgG Response to the *P. falciparum* CSP Protein

**Figure 2:**
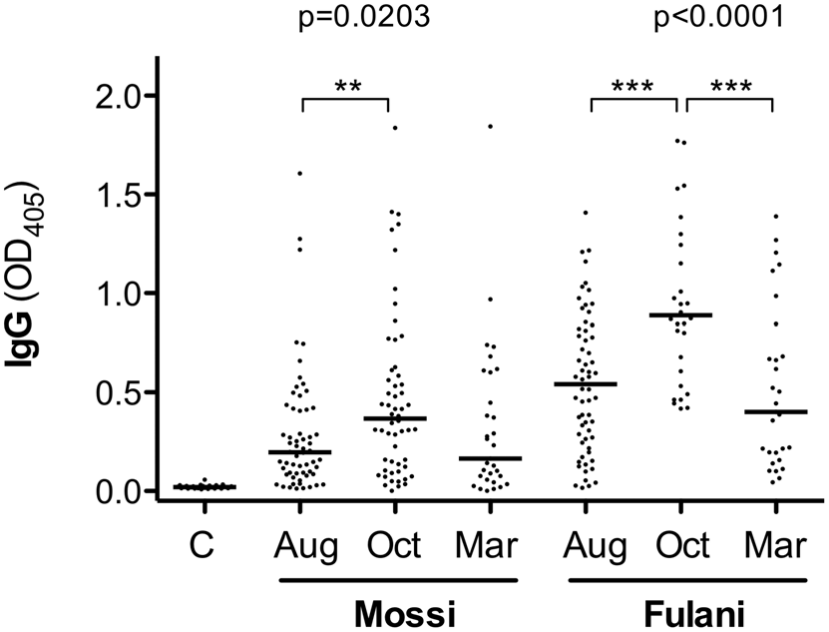
Seasonal variation of the IgG response to the *P. falciparum* CSP in Mossi and Fulani. Scatter plot of OD values representing the IgG response to the *P. falciparum* CSP among gSG6 IgG responders of the Mossi and Fulani ethnic groups in the three different surveys. C, unexposed controls (n = 28). Bars, number of Mossi and Fulani analyzed, p values and pairwise comparisons as in Figure 1. Note that three data points are outside the axis limits.

We are also reporting here, as comparison, the IgG antibody response to the *P. falciparum* CSP in the same group of anti-gSG6 IgG responders. As expected, the IgG response to CSP showed seasonal variation in both ethnic groups (Kruskal-Wallis test: Mossi p = 0.0203, Fulani p<0.0001) with a significant increase during the transmission season (August vs October, Mann-Whitney U test: Mossi p = 0.0076, Fulani p<0.0001) and a decrease during the dry season that was significant in the Fulani (October vs March, Mann-Whitney U test, p = 0.0002) (Figure 2). Moreover, IgG levels were always higher in the Fulani as compared to Mossi (Mann-Whitney U test: August p<0.0001, October p<0.0001, March p = 0.0236), a result fully consistent with what previously reported [26]. No correlation was found between the IgG response to the CSP, which is supposed to denote exposure to *Plasmodium* sporozoites (i.e. to infectious mosquito bites), and IgG1 or IgG4 responses to the gSG6 salivary protein, which is expected to reflect overall mosquito exposure.

### 3.3. Humoral Response to the gSG6 and CSP Proteins According to Age

**Figure 3:**
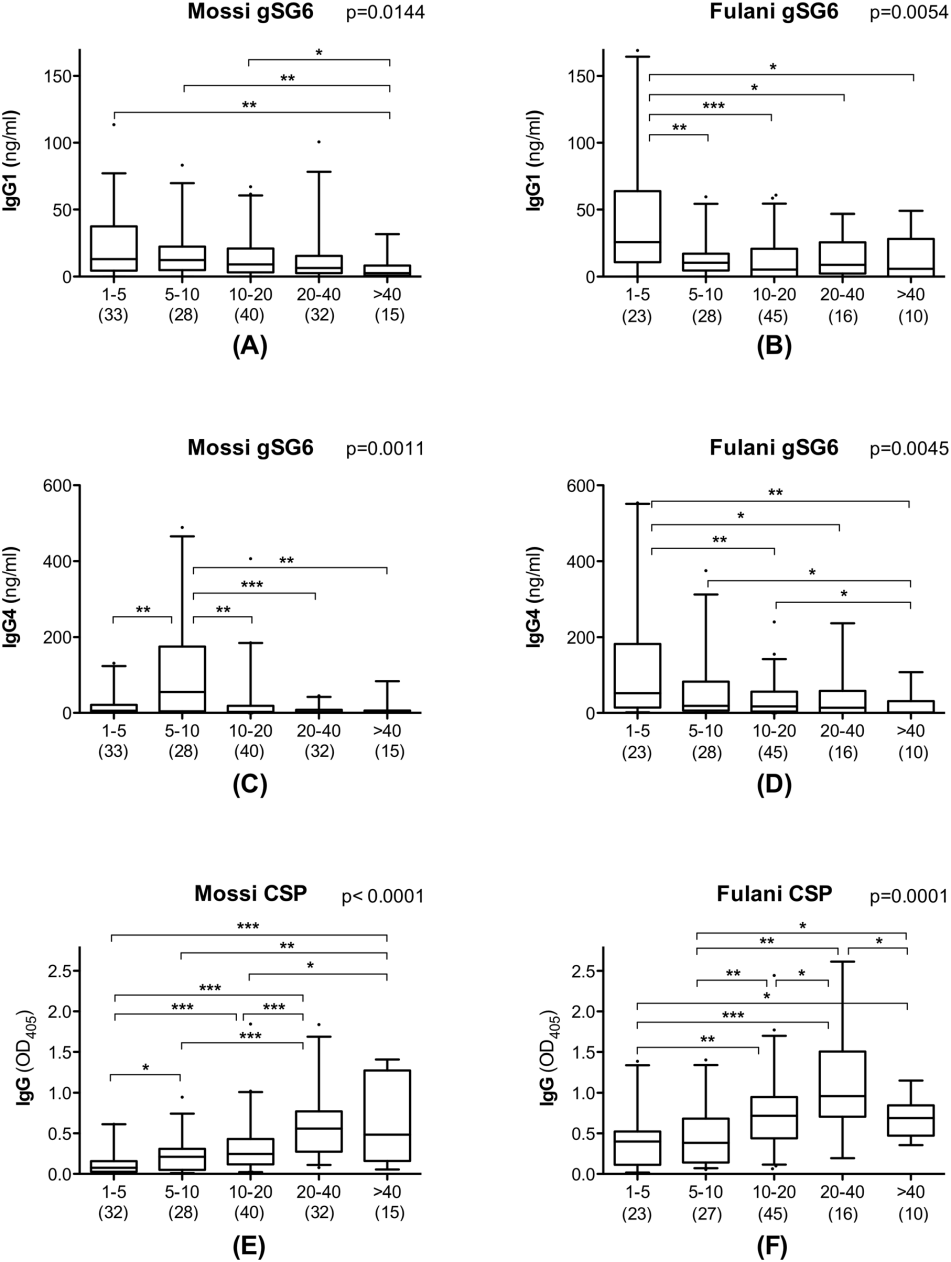
Distribution of anti-gSG6 IgG1 and IgG4 and of anti-CSP IgG in Mossi and Fulani according to different age groups. Box plots showing IgG1 and IgG4 response to gSG6 (A-D) and IgG response to CSP (E-F) among gSG6 IgG responders from the three different surveys. Boxes display median values, 25^th^ and 75^th^ percentiles. Whiskers represent 5–95 percentiles and dots the outliers. Anti-gSG6 IgG1 and IgG4 antibody titers are expressed in ng/ml, anti-CSP IgG levels as OD_405_. The five different age groups (years) are indicated at the bottom. Data from the three different surveys were pooled and the number of individuals for each age group is given in parenthesis. Left panels (A, C, E) refer to Mossi and right panels (B, D, F) to Fulani as indicated. P values were determined by the Kruskal-Wallis test and pairwise comparisons were according to Mann-Whitney U test (*, 0.01<p<0.05; **, 0.001<p<0.01; ***, p<0.001).

**Figure 4:**
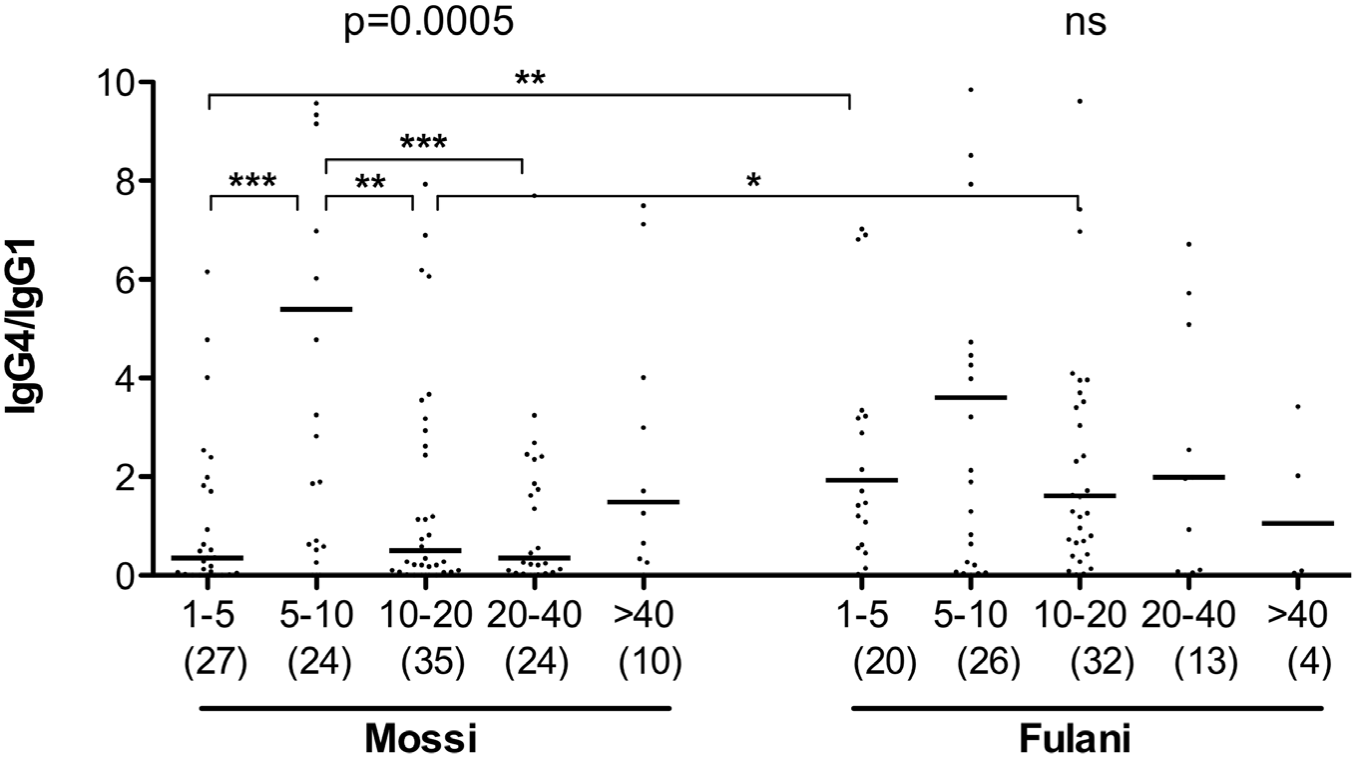
IgG4/IgG1 ratio by age group in Mossi and Fulani. Scatter plot reporting the IgG4/IgG1 ratio by age group in Mossi (n = 120) and Fulani (n = 95). Data from the three different surveys were pooled together and individuals with no detectable IgG1 or IgG4 (or both) were excluded from the analysis. The five different age groups (years) are indicated at the bottom. The number of individuals for each age group is given in parenthesis. Bars indicate median values. P values determined according to the Kruskal-Wallis test. Pairwise comparisons refer to the Mann-Whitney U test (*, 0.01<p<0.05; **, 0.001<p<0.01; ***, p<0.001). Note that twenty data points are outside the axis limits.

In a previous investigation on a larger set of samples including the ones analyzed here we found, both in the Fulani and in the Mossi, a progressive decrease with age of the anti-gSG6 IgG response [18]. When the subset of IgG responders studied here was sorted in five different age groups (1–5, 5–10, 10–20, 20–40 and >40 years old) we observed a similar situation for the IgG1 and IgG4 responses to gSG6. As a general trend, median levels of gSG6-specific IgG1 and IgG4 antibodies appeared to decrease with age progression: this applied both to Mossi and to Fulani, although statistical support was only obtained for the August survey (not shown), perhaps also because of its slightly larger and better balanced sample size. However, when data from the three surveys were pooled and analyzed together, the age decrease was statistically significant in Mossi and Fulani both for IgG1 and IgG4 (Kruskal-Wallis, 0.0144≥p≥0.0011, Figure 3), although with a slightly different pattern in the two ethnic groups. Actually, median IgG1 titers had their peaks in 1–5 years old children in both groups (Figure 3A–B), whereas median IgG4 titer also peaked in young children in the Fulani but later, at 5–10 years of age, in the Mossi (Figure 3C–D). The pattern appeared even more clear looking at the IgG4/IgG1 ratio, with comparable values among the five age groups in the Fulani and a significant difference in the Mossi (Figure 4; Kruskal-Wallis, p = 0.0005). At 1–5 years of age the ratio was significantly higher in the Fulani as compared to the Mossi (median values: Mossi 0.35; Fulani 1.93; Mann-Whitney U test, p = 0.0019), whereas no difference was found between the two tribes in 5–10 years old children (median values: Mossi 5.40; Fulani 3.60). These observations suggest that the humoral response to the gSG6 salivary protein involves a switch from IgG1 to IgG4 which takes place earlier in the Fulani (before 5 years of age) and only later in the Mossi (in 5–10 years old children). After 10 years of age the ratio IgG4/IgG1 decreases slightly in the Fulani but drops significantly in the Mossi (Mann-Whitney U test, p = 0.0012) and in 10–20 years old individuals the ratio appears again significantly higher in the Fulani (median values: Mossi 0.50; Fulani 1.61; Mann-Whitney U test, p = 0.0259). After reaching the age of twenty the IgG4/IgG1 ratio does not seem to change significantly anymore in the two ethnic groups, although it should be noted that in the last age groups the lower sample size makes the statistical analysis less robust.

**Figure 5:**
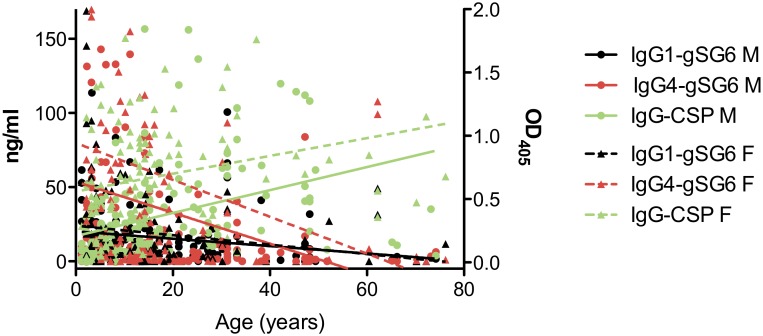
Humoral response to the gSG6 and CSP proteins according to age. Scatter plot reporting the antibody responses to gSG6 (IgG1, black; IgG4, red) and to CSP (IgG, green) as function of age among gSG6 IgG responders of the Mossi (n = 148) and Fulani (n = 122) ethnic groups from the three different surveys. Anti-gSG6 IgG1 and IgG4 are expressed as titers (ng/ml, left Y axis); IgG response to CSP is expressed as OD_405_ (right Y axis). The best-fit lines are shown (Mossi, solid lines; Fulani dashed lines). Spearman correlation coefficients: (i) anti-CSP IgG (Mossi, r = 0.53, p<0.0001; Fulani, r = 0.41, p<0.0001); (ii) anti-gSG6 IgG1 (Mossi, r = −0.25, p = 0.0019; Fulani, r = −0.27, p = 0.0024); (iii) anti-gSG6 IgG4 (Mossi, r = −0.12, p = 0.1359; Fulani, r = −0.29, p = 0.0013). Note that twenty-six data points are outside the axis limits.

As expected the response to the *P. falciparum* CSP follows the typical pattern already known for this and other parasite antigens, i.e. an increase from childhood to adulthood, with a slight decrease in elderly people in both ethnic groups (Figure 3E–F). The different trend in the response to the mosquito gSG6 and to the parasite CSP antigens is well summarized by the scatter plot shown in Figure 5 where the anti-gSG6 IgG1 and IgG4 titers and the anti-CSP IgG response are reported in function of age. The best-fit lines show the strong correlation with age of the IgG response to CSP (Mossi: Spearman r = 0.53, p<0.0001; Fulani: r = 0.41, p<0.0001), whereas an inverse correlation was found in both ethnic groups for the IgG1 (Mossi: r = −0.25, p = 0.0019; Fulani: r = −0.27, p = 0.0024) and IgG4 (Mossi: r = −0.12, p = 0.1359; Fulani: r = −0.29, p = 0.0013) responses to gSG6. Moreover, the trends depicted by the best-fit lines in Figure 5 show that, independently from the age, the IgG1 response to gSG6 is similar in the two tribes whereas both the anti-gSG6 IgG4 response and the IgG response to CSP are higher in the Fulani.

## Discussion

We measured the anti-gSG6 IgG1 and IgG4 antibody levels in the sympatric ethnic groups Mossi and Fulani and found that IgG1 and IgG4 titers were high during the rainy season and decreased during the dry season in the Mossi. This was not surprising since a change of gSG6-specific IgG antibody levels according to malaria transmission and vector density was also observed in individuals of the less protected Mossi group in a previous study [18]. Moreover, similar seasonal variations of IgG1 and IgG4 response to mosquito saliva were previously reported in a Finnish group exposed to *Aedes communis* [29]. By contrast no seasonal variation of the anti-gSG6 IgG1 and IgG4 antibody titers was observed in individuals of the more protected Fulani group. We hypothesized that the higher baseline level of immunity in the Fulani may mask any temporal variations of the anti-gSG6 humoral response and the results reported here seem to further support this interpretation.

Individuals of the Fulani ethnic group were previously found to carry higher titers of circulating IgG antibodies against the salivary protein gSG6 as compared to Mossi [18]. Here the two tribes showed very similar titers of anti-gSG6 IgG1 antibodies during the rainy season; only during the dry season, when titers dropped in the Mossi while they stayed essentially unchanged in the Fulani, IgG1 levels were higher in the latter. A different pattern was found for IgG4 antibodies: in all the surveys median anti-gSG6 IgG4 titers were higher in the Fulani and the difference was significant both during the rainy (August) and the dry (March) periods. Human IgG response to mosquito bites is known for being mainly characterized by saliva-specific antibodies of the IgG1 and IgG4 subclasses [35]–[37]. We do not know if the gSG6 protein may also evoke high levels of IgG2 and IgG3 antibodies, nevertheless the observations reported here suggest that IgG4 antibodies may account for most of the difference in the anti-gSG6 IgG response between these two ethnic groups. We should emphasize here that, although exposure to *Anopheles* mosquitoes was rather high in both villages during the transmission season (from 6.3±1.5 to 21.8±10.3 *Anopheles*/person/night in the period August–October), vector density was slightly higher in Barkoundouba, which is inhabited by the Fulani [18]. Moreover, it may be argued that also known behavioral differences such as cattle herding or guarding may potentially result in higher exposure of Fulani to outdoor biting as compared to Mossi. For these reasons we cannot rule out the possibility that a difference in mosquito biting rates may contribute to the higher anti-gSG6 IgG4 titers reported here. Nevertheless, our findings suggest that the genetic diversity between Fulani and Mossi is playing a key role in their different immune response to mosquito salivary antigens, which is fully consistent with the different response of these two ethnic groups to malaria antigens [26], [27], [30]–[34]. In this respect, earlier studies on the response to parasite antigens of Mossi and Fulani, either from Mali or from Burkina Faso, showed that IgG4 titers were usually very low with IgG1 and IgG3 being the dominant IgG subclasses [30]–[32]. Only one study reported higher IgG4 titers against the *P. falciparum* 3D7-MSP2 allele in a Fulani group from Eastern Sudan as compared to non-Fulani neighbors [34]. We found here high levels of IgG4 antibodies against the gSG6 protein in both groups, with IgG4 titers higher in the Fulani than in the Mossi. Moreover, in the Fulani IgG4 titers were significantly higher than IgG1 titers in all the surveys, pointing to IgG4 as the dominant circulating antibody subclass against the gSG6 protein; this was not the case for the Mossi where no difference between anti-gSG6 IgG4 and IgG1 titers was found.

High levels of antigen-specific IgG4 are most likely associated to the continued exposure of the population under study to *Anopheles* saliva and, perhaps, to allergen-like properties of the gSG6 protein. Moreover, the increase of the anti-gSG6 IgG4/IgG1 ratio may be related to the development of tolerance to mosquito salivary components. Indeed it is known since the mid-twentieth century that intense, repeated and prolonged exposure to mosquito bites may induce desensitization, with disappearance of immediate and delayed cutaneous reactions [38]–[40]. Furthermore, earlier longitudinal studies on beekepers clearly showed that prolonged exposure to honey bee venom induces a shift in the lgG4/lgG1 antibody ratio. Indeed, in novice beekeepers IgG antibodies to phospholipase A2 (PLA2) are predominantly of the IgG1 subclass; however, the relative contribution of lgG4 to total anti-PLA2 IgG response increases with time and virtually all beekeepers with an history of exposure of more than 3 years exhibit an anti-PLA2 IgG4-dominated response [41]. Similarly, higher levels of food-specific IgG4 have been associated with tolerance in food atopic children [42], [43].

It has been previously suggested that the age decrease of the anti-gSG6 IgG response may be ascribed to the development of immune tolerance [18]. The shift of the anti-gSG6 IgG4/IgG1 antibody ratio found here fully supports the idea that the continued and intense exposure to *Anopheles* bites induces desensitization to mosquito salivary components in the population under study. As mentioned above for beekepers, allergen-specific IgG4 usually appear only after prolonged immunization and their presence is considered as a sign of activation of anti-inflammatory and tolerance-inducing mechanisms [44]. Accordingly, the appearance of allergen-specific IgG4 antibodies has usually a protective effect and is associated with an improvement in allergic symptoms, most likely due to their capacity to compete for allergen binding with cell surface-bound IgE, which prevents activation of mast cells and inhibits antigen-presenting cells [45]. The mechanisms inducing tolerance against mosquito bites are presently not fully understood but are likely to involve Treg cells, which play a key role in maintenance of immune tolerance and down-regulation of inflammatory responses, also through their secretion of IL-10 and TGF-β [46]. IL-10 in particular affects class switch and enhances IgG4 production [44], [45] suggesting a possible involvement of IL-10-producing Treg cells in the immune response to the gSG6 antigen. Indeed a higher frequency of CD4+ CD25+ IL-10-secreting cells was recently reported in the Fulani ethnic group as compared to Mossi in the same area of Burkina Faso [47], although in a previous study similar levels of IL-10 were found in the sera of 58 Fulani and 82 Mossi collected during the same August 1994 survey from the Barkoundouba area analyzed here [27].

Taken together our data show that the gSG6 antigen carried in the saliva of anopheline mosquitoes induces in exposed individuals a T helper type 2 immune response with production of IgG1 and IgG4 antibodies. Moreover, the intense and prolonged exposure to *Anopheles* bites induces the activation of mechanisms of immune tolerance with antibody class switch and production of high levels of IgG4. Further studies will be needed to elucidate the mechanisms determining both higher levels of circulating anti-gSG6 IgG4 antibodies and the earlier switch from IgG1 to IgG4 observed in individuals of the Fulani ethnic group. In conclusion the study reported here on the *An. gambiae* gSG6 represents a first step toward a better understanding of the human immune response to mosquito salivary proteins, a subject which would deserve more attention, especially considering that mosquitoes are vectors of devastating viral and parasitic diseases and that these pathogens are transmitted and exposed to the human immune system in the context of mosquito saliva.
